# The home language environment in rural China: variations across family characteristics

**DOI:** 10.1186/s12889-023-15245-2

**Published:** 2023-02-16

**Authors:** Yue Ma, Laura Jonsson, Zixin Yao, Xinwu Zhang, Dimitris Friesen, Alexis Medina, Scott Rozelle, Lucy Pappas

**Affiliations:** 1 Rural Education Action Program, Freeman Spogli Institute for International Studies, Stanford University, Palo Alto, CA USA; 2 Economics Department, Columbia University, New York City, USA; 3 School of Public Administration, Northwest University, 1 Xuefu Road, Chang’an District, 710127 Xi’an, Shaanxi PR China

**Keywords:** Early childhood development, Home language environment, Language development, urban/rural differences

## Abstract

**Background:**

A rich language environment is an important element of a nurturing home environment. Despite their proven importance, vocabulary and conversation have been shown to vary widely across households—even within the same socio-economic class. One significant gap in the existing literature is its nearly exclusive geographic focus on Western and developed settings, with little attention given to poorer communities in lower/middle income countries. The purpose of this study was to empirically illustrate the characteristics of the home language environment in the low SES, non-Western cultural setting of rural China.

**Methods:**

Using Language Environment Analysis (LENA) automated language-analysis system, this study measured the home language environment of 38 children aged 20-27 months in Northwest rural China. Our primary measures of the home language environment were Adult Word Count (AWC), Conversational Turn Count (CTC) and Child Vocalization Count (CVC). Multivariate linear regression models were used to examine the association between home language environment and family/child characteristics, and language skills (Measured by MacArthur-Bates Communicative Developmental Inventory score).

**Results:**

In this paper, by comparison, we found that the home language environment of our rural sample fell far behind that of urban households. We also identify significant, positive correlations between language skills and both AWC and CTC. Our analysis finds no significant correlations between home language environment and family/child characteristics.

**Conclusion:**

In this paper, we present the first ever findings using the LENA system to measure the home language environment of young children from poor rural communities in China. We found that the home language environment of lower-SES household was significantly worse than high-SES households, and demonstrated the importance of the home language environment to language skills, pointing to a need for more high-quality studies of the home language environment in rural China to better understand possible mechanisms behind low levels of parent-child language engagement and ways to improve the home language environment.

**Supplementary Information:**

The online version contains supplementary material available at 10.1186/s12889-023-15245-2.

## Introduction

An estimated 250 million children (43%) under 5 years in low- and middle-income countries (LMICs) are at risk for reduced cognition and developmental delays [1, 2]. An essential component of child development, early language development is considered by experts to be a useful indicator of a child’s cognitive ability [3], brain development [4], and is related to later school success [5, 6]. Early childhood is a crucial period for children in developing settings where language delays are prevalent [7–9]. In these first few years of life, a variety of factors influence language development, including nutrition, health and poverty[10, 11]which are often lacking in developing countries [12].

High prevalence rates for language delay have been reported in LMICs. Mondal et al. (2016) examined 200 children, less than three years old in India, and found that the prevalence of speech and language delay was 27% [13]. Chunsuwan et al. (2016) examined 266 children aged 9, 18 and 30 month in Thailand and reported that expressive language is the most common delayed domain (19.2%) [14]. Dias et al. (2020) reported that the prevalence of language delay was 12.5% among 1000 children aged between 0 and 5 years in Brazil [15]. As China is one example of a low-socioeconomic status (SES), LMIC setting, and yet over 40% of residents live in largely underdeveloped, rural areas [16]. As compared to urban areas, China’s rural areas are characterized by disparities in education [17], income [18], and human capital [19]. Children in rural China suffer serious disadvantages: young children living in these areas have been consistently shown to have high rates of delays in their language skill development [7–9, 20]. In terms of general delays, 85% of children under three years old in rural China were found to have a developmental delay, and over half had delayed language skills in one large-scale study [8]. Evidence indicates that untreated speech and language delay in preschool children can persist in 40–60% of the children and these children are at a high risk for social, behavioral, emotional, and cognitive problems in their later years [21].

Research shows that many factors may affect language ability, including the home language environment [22]. The literature consistently shows that the more parents speak to their children, the faster the children’s vocabularies grow, and the higher they score on cognitive skill scales at age three years and beyond [23–25]. Indeed, the child’s home language environment has been identified as one of the main determinants of early language skills [26, 27]. Early language skills, in turn, have been shown to be predictive of academic success or failure later in childhood [28, 29].

Given the significance of a rich home language environment, the LENA (Language ENvironment Analysis) system provides a convenient method for measuring this environment. LENA presents an objective and quantitative characterization of language environments by using measures like adult word count (AWC), conversational turn count (CTC), and child vocalization count (CVC). The AWC means the number of adult words spoken, estimate of number of words from adults that were spoken to and near the key child; the CTC was defined as adult-child alternations per day, as the child says something and the adult responds within 5 s or vice versa; and CVC is defined as the frequency of child speech related events such as sounds, words, or vocalizations that were not vegetative noises, cries, or coughs[30]. Past research relied on bulky and invasive home video recordings to observe parent–child talk [23, 31], which may not have simulated the home environment and could be cumbersome and required extensive time for analysis [32]. Also, the costs and logistics associated with these methodologies might be particularly unwieldy [32]. LENA is a new tool created to address these issues by combining a wearable audio recorder with automated vocal analysis software [33]. Using LENA, numerous studies have confirmed the fundamental role that the quantity (AWC) and quality (CTC) of early interactions play in infants’ early and later language and general cognitive development [26, 34–36]. Additionally, the rates and durations of AWC and CTC have been found useful in characterizing the language environments of children with barriers to language development, like hearing deficits and language delays, and can differentiate between those children from typically developing children [37, 38]. The importance of CVC has been noted in the development of pre-term infants, as parental-talk is a strong predictor of infant vocalizations [39]. These advantages have led to LENA’s widespread use, as more than one hundred studies have used this system over the past two decades [40].

By using the LENA system, previous research has shown several correlations between family characteristics and the home language environment. Family income and parental education play large roles in determining the home language environment, as children from high-SES families have been shown to have richer home language environments than those from low-SES families [26, 27, 41, 42]. For example, low-SES mothers have been found to talk less and use less-varied vocabulary during interactions with their children than do high-SES mothers [43]. Additionally, early talk and interaction, particularly between 18 and 24 months, can predict school-age language and cognitive outcomes [35].

Although the LENA system has been used to generate a wealth of evidence about the nature of the home language environment and language skill development, one significant gap in the existing literature is its nearly exclusive geographic focus on Western and developed settings. In our literature review, while we found a plenitude of LENA research on the home language environment in Western and developed settings [26, 30, 34, 37, 41, 43–49], we found significantly fewer studies that explored this topic in non-Western settings [32, 50–54]. These studies present high-quality research in several non-Western and developed settings, however each of these settings is quite unique and widely distributed, spanning from East Asia to Africa. As a result, the relative scarcity of LENA research in non-Western settings is impeding the discovery of overarching trends in these areas.

Of this small handful of studies focusing on non-Western settings, few focus on Asian settings [32, 50, 52–54], and even less focus on China [52, 54]. In South Korea, Pae et al.(2016) found that in families with worse linguistic environments, AWC and CTC were significantly lower [50], while the studies from Vietnam [32, 53] focused on a validation protocol and a comparison between Vietnamese and Canadian families, finding that Canadian families participated in more conversational turns. In China specifically, Zhang et al. (2015) explored the variations of LENA measures among urban families and their correlations with child development measures while providing quantitative linguistic feedback to caregivers [52]. They found that among LENA measures, AWC and CTC improved significantly over the first three months of the intervention but returned to baseline after six months, with families in the lower 50% at baseline accounting for most of these changes. Zhang et al.(2015) also found that CTC was positively correlated with language skills development scores (measured by Mac-Arthur Bates Communicative Developmental Inventory) after three months of the intervention [52].

This presents a notable gap in the literature. In rural China, interactive parenting (i.e. playing, singing and telling stories) has been identified by the literature as strongly linked to developmental delays, especially in the case of language delays[7–9]. Previous research has included simple measures of the home language environment in rural China, however these studies only use qualitative, self-reported measures, like the Family Care Indicator [7–9]. This measure, despite producing important data like how often parents read to their children, sing to their children, play games with their children and how long they spend with their children, is still self-reported from caregivers, and is potentially subjective.

The overall goal of this study is to objectively and quantitatively assess the home language environment in a poor area of rural China using the LENA system. To do so, we have two objectives. The first objective is to describe the rural home language environment and to compare these results to LENA data from comparable studies conducted in urban China. The second objective is to identify and discuss family characteristics associated with differences in the rural home language environment, as well as to show correlations between the home language environment and language skills.

In the absence of evidence to the contrary, our ex-ante hypothesis is that our sample from rural China will largely follow trends consistent with those found in the international literature. Specifically, we expect that family factors such as SES, parental education, identity of the primary caregiver, number of siblings, and parental migration will all be correlated with the home language environment [26, 27, 41, 42, 45, 52, 55]. We also expect to see positive and significant correlations between the home language environment and child language skills [26, 34–39, 52].

## Methods

### Sample selection

The data for this study were collected in 2019 from five counties with relatively low levels of economic development in Shaanxi Province, geographically situated in northwest China. A mixture of the Shaanxi dialect and Mandarin (SDM) languages is spoken in Shaanxi Province. The Shaanxi dialect has been established as a Mandarin-based dialect that bears a very close linguistic similarity to Mandarin [56]. Moreover, the Shaanxi dialect can best be thought of as Mandarin with a slight accent—the grammatical structure is virtually identical [56].

Our target population was households with children aged 20–27 months. We selected this age range because it is an important period in language development, and is the stage in which children begin to accelerate their vocabulary acquisition [35]. We formulated our sampling protocol accordingly.

The research team followed a three-step protocol to choose households within the sample counties. First, out of all of the townships in the counties, one township was randomly selected from each county. Second, the team randomly selected one village from each township to participate in the study. If there were too few (< 8) children aged 20–27 months (the desired age range), the research team randomly selected additional villages from the same township until we had selected at least 8 children per township. Third, all of the children in the desired age range were included in the sample and invited to participate in the study. In the five study counties and townships, the sample included 38 families with young children from 16 villages.

### Data collection

For each child in the sample, we collected LENA measurements of their home language environment and conducted a survey, that was designed to collect information on both child and family characteristics, with their caregiver. The LENA collection protocol is described below. On the day after the LENA recordings were completed, the research team administered the survey collecting child and family characteristics as well as information regarding each child’s language skills (through a parent-reported scale).

#### Measure of the home language environment: the LENA system

Language environment measures were obtained using the LENA system [57, 58]. A small digital recorder was worn by the child in the front chest pocket of specially tailored clothing designed to optimize microphone placement and minimize friction-based noise. Recorders capture16 h of high-quality audio data, which is optimally recorded within a 6 to 10-foot radius at 16 kHz. Completed recordings were processed by LENA software to produce the three metrics used in this study: AWC, the number of adult words spoken, estimate of number of words from adults that were spoken to and near the key child; CTC, adult-child alternations per day, as the child says something and the adult responds within 5 s or vice versa; and CVC, counts of chunks of speech-related sounds produced by the key child. LENA has previously been validated to be reliable in many languages [32, 50, 59–62], including Mandarin Chinese [52, 60].

Each family was asked to produce one LENA recording to estimate the home language environment. The recording was scheduled to be completed on what the household described as a “normal day^1^.” In rural China, caregivers typically stay with the child at home for most of the time during a “normal day” as full time caregivers. Members of the research team delivered the LENA recorders at 9 am on the morning of the first day. Before the team left the household, they made sure that the child was wearing their LENA-designed clothing (vest or coverall) and that the recorder was turned to the “record” position. The research team asked parents to keep a log of the locations in which the recording was conducted, who was present, the main activities the child was engaged in, and whether anything atypical occurred during the day. The research team then picked the recorder up the following afternoon, on day two. Families were instructed to only remove the recorder when the child bathed and went to sleep for the night. Before collecting the recorders from participating families, our trained enumerators confirmed that the recorded day was accurately representative of normal life. If the parents indicated that the recording day was atypical (e.g., the child was sick), then the family was asked to redo their recording to make sure that the recording was representative.

Due to variations in the recording starting times between families, our 16-hour recordings were standardized into 12-hour segments. The main outcomes of our LENA results are normally distributed (Appendix Figs. 1, 2 and 3 show the kernel density plot distribution of each language outcome), and thus can be adjusted following the procedure described below. We adjusted our data to account for common skewing. We first normalized the distribution of data via Chebyshev polynomials transformation. We then selected the final model via LASSO regression models. Third, the final Chebyshev polynomials model was used to predict the residuals. The transformed data was used to estimate residualized count variables, which were then rescaled back to the original count metric. Official outcomes were AWC, CTC and CVC totals from the first usable 12-hour recordings of the participants.

**Fig. 1:**
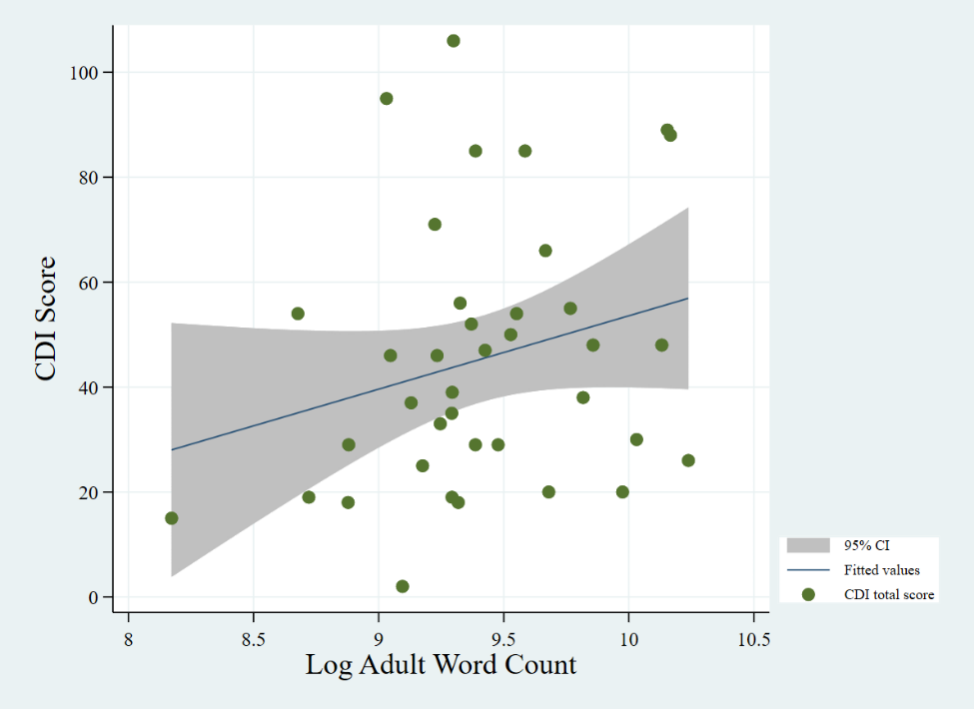
Correlation between AWC and CDI (P-value: 0.027)

**Fig. 2:**
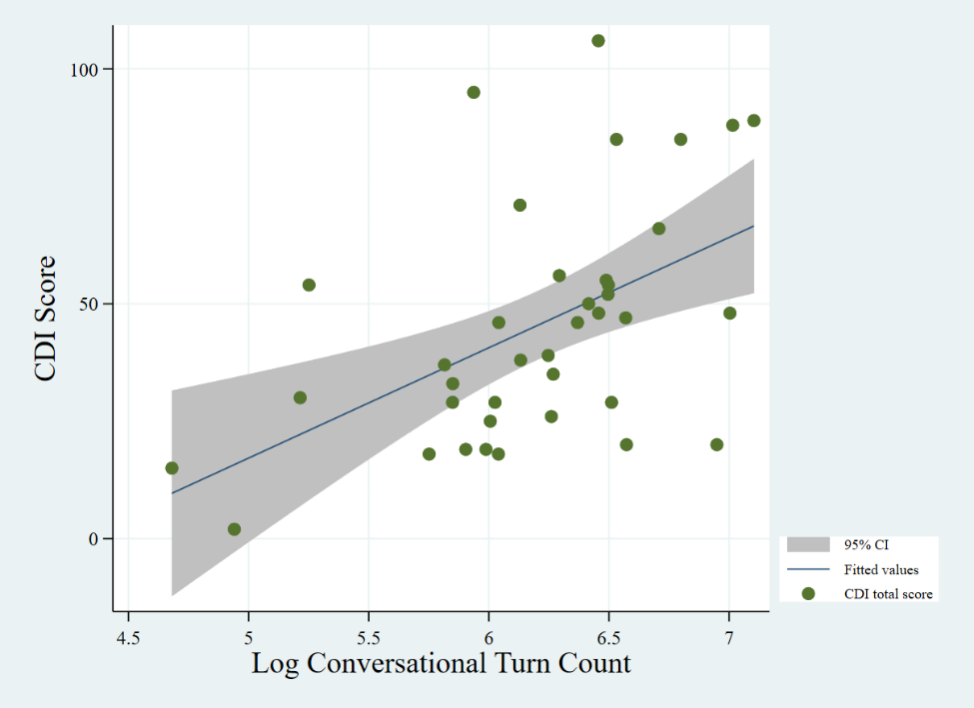
Correlation between CTC and CDI (P-value: 0.002)

**Fig. 3:**
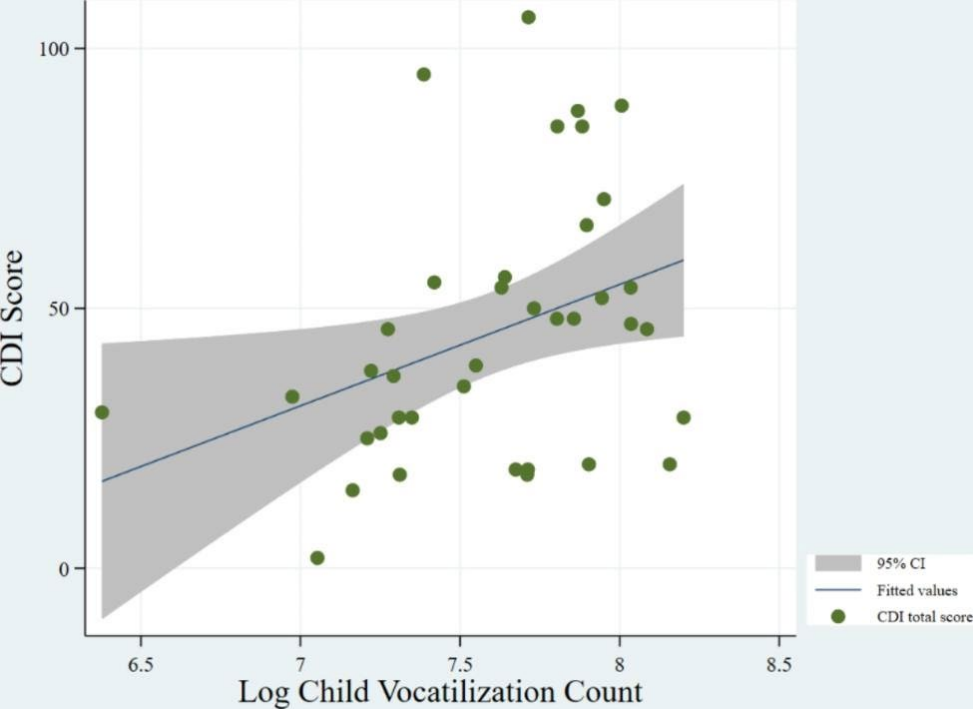
Correlation between CVC and CDI (P-value: 0.072)

To evaluate the performance of the LENA automated language-analysis system for the Shaanxi dialect and Mandarin (SDM) Chinese languages, we randomly selected 13 households from 38 households for the current validation analysis. Next, we selected a minimum of three 5-minute audio segments per family, representing periods of high, medium, and low interaction. In sum, we extracted three 5-minute audio samples for each of 13 families (195 min, or 3.25 h, total). To derive accurate rater-based AWC, CTC, and CVC from these 5-minute audio samples, a native Chinese speaker (who was blinded to the LENA results) completed the hand transcriptions and analysis of the audio samples, following LENA validation protocols (see Appendix Protocol) from previous studies [60, 63].

Owing to SDM Chinese being tonal with a prosody and containing a number of phonemes different markedly from that of English [60], for AWC, the rater annotated each segment identified as containing clear adult speech with two count values: the number of discrete Chinese characters and the number of Romanized pinyin words by which adult speech in the segment could be represented. Chinese characters are not letters but rather are a form of pictogram. In most cases, each character is equivalent to one spoken syllable, and spoken SDM words typically comprise between one and four syllables. The rater followed standard word-formation rules in grouping characters to derive word boundaries and thus counts (see http://www.pinyin.info/readings/zyg/rules.html). For conversational turns, CTC includes only back-and-forth interactions between the child wearing the recorder and an adult in his/her environment. The rater counted the number of back-and-forth interactions between the child wearing the recorder and an adult in the environment following the rules of the Appendix Protocol (i.e., vocal alternations occurring between adult and child within 5 s, uninterrupted by other speaker segments). Regarding CVC, “child vocalization” estimates the number of any speech-like babbling or vocalizations within a child utterance cluster. The rater also counts the number of speech-like babbling or vocalizations of the child wearing the recorder following the rules of the Appendix Protocol (e.g., the child said “ma” or “mamamama” this was counted as one vocalization.).

The reliability and validity of LENA segmentation and AWC, CTC, and CVC estimates for SDM-speaking families were assessed via comparisons with human rater values. We used Stata 16.1 to obtain descriptive statistics, correlations, and means comparisons by paired-samples t tests. Results are shown in Appendix Table 1. Chinese character counts were highly and significantly correlated with pinyin word counts, r (13) = 0.65, p < 0.001, and the correlations of each with AWC were reasonably high and statistically identical. Consistent with Gilkerson et al. (2015), AWC was significantly different from Chinese character counts [60]. However, SDM word counts were not significantly different from AWC, demonstrating that AWC provided a reasonably accurate estimate of adult SDM speech. For CTC, no mean differences were observed between CTC and conversation turns on the basis of rater segmentation, as the two values were highly and significantly correlated. We also found the same results between CVC and child vocalization on the basis of rater segmentation. Thus, LENA AWC, CTC, and CVC provided reasonably accurate estimates for the SDM languages.

**Table 1:** Descriptive statistics of family characteristics

Variables	Rural northwestern China sample (N = 38)
***Child characteristics***	
Age in months, Mean (SD)	24.58 (2.07)
Male, n (%)	20 (52.63)
***Family characteristics***	
Age of mother in years, Mean (SD)	28.76 (3.66)
Mother completed middle school or above, n (%)	25 (65.79)
Mother is the primary caregiver, n (%)	28 (73.68)
Number of adults in the household, Mean (SD)	2.08 (1.08)
Whether there are siblings at home, n (%)	10 (26.32)
Father completed middle school or above, n (%)	20 (52.63)
Father lived at home during most of the last year, n (%)	24 (63.16)
Asset index (PCA score), Mean (SD)	0.00 (1.77)

Source: Authors’ survey.

Note: In our survey, the education level of both the mother and father is recorded as a binary variable equal to 1 if that parent completed middle school or above, or 0 if they did not. Completion of middle school requires 9 years of total schooling and is the last stage of compulsory/free education in China.

#### Language skill measurement

To measure a child’s developing abilities in early language (i.e., vocabulary comprehension, production, gestures, and grammar), we used the Mandarin version of the MacArthur-Bates Communicative Development Inventories (CDI), a parent-report assessment which has been adapted and validated in Mandarin Chinese[64, 65]. Past studies have used this assessment and proven its reliability in studying early childhood language development in China [52, 66]. We utilized the expressive vocabulary assessment of the CDI for children between 16 and 30 months (our participating children were 20–27 months old). Using a list of 113 words, enumerators asked the child’s primary caregiver whether their child could say each word; each word the child could say counted for one point. When administering the CDI, all primary caregivers were periodically asked to provide an example of when they observed their child using the particular word. The procedure of conducting CDI was followed exactly from Fenson et al. (2007), and questions were read by members of our research team to the caregivers[65].

#### Demographic information

For each child, we recorded their sex and exact age in months. The survey also collected information on family characteristics, including mother’s age, maternal education level, paternal education level, whether the father lived at home during most of last year, the child’s primary caregiver (mother or others), the number of adults in the household, the number of siblings in the household, and family assets. For the family assets, we established a family asset index for participating households using polychoric principal components analysis (PCA) (a dimensionality-reduction method that creates a visualization of data that minimizes residual variance in the least squares sense and maximizes the variance of the projection coordinates) based on whether the family owned or had access to running water, a flush toilet, a water heater, a washing machine, a computer, Internet, a refrigerator, an air conditioner, a motorbike/motorcycle, and a car/truck [67].

Our focus on these particular indicators is rooted in the literature: Child age and sex were collected because many studies have found differences in language development between girls and boys [9, 52] also that older children have better language skills [35]. Many parental characteristics related to socioeconomic status, including parental age, parental education, and parental migration status, have also been shown to be associated with language development [26, 27, 68]. Household economic status has been shown to be associated with early childhood development as well [9, 35]. Identity of the primary caregiver was collected because previous research has shown that about one-third of primary caregivers of young children in rural China are actually grandmothers, not mothers, and that caregiver type affects early childhood development outcomes [9]. Finally, the numbers of adults and siblings in the household were collected as it has been suggested that household size is an influential factor in the home language environment and language development [69, 70].

To determine whether the inclusion of these demographic information variables is valid in our study, we created Kernel density plots to test the distribution of the continuous covariates and have analyzed the variances of the binary covariates. We find that all continuous covariates are normally distributed, and that the variance of all binary covariates are appropriate for use in our analysis. Thus, we feel justified in our inclusion of all demographic variables in our analysis.

### Statistical analysis

All statistical analyses were performed using Stata 16.1. P-values at or below 0.05 were considered statistically significant. In our multivariate simple linear regressions of the home language environment, in addition to presenting average outcomes for our full sample, we present outcomes for the top half and bottom half of the sample separately (above and below the median level). To control for the family-wise error rate, in our multivariate simple linear regression analysis, we use the Bonferroni Correction to adjust the α value used to assess significance (αnew = αold / n) [71]. The new α value used is *p < 0.005. In the multivariate multiple linear regressions, we include the following variables as potential covariates: child’s age in months, child’s sex, mother’s age, maternal educational level, paternal education level (both parental education levels are measured as a binary variable with the variable equaling 1 if the parent completed middle school or beyond), whether father lived at home during most of last year, the identity of the child’s primary caregiver (which was measured as a dummy variable with the variable equaling 1 if the caregiver was the child’s mother), number of adults in the household, number of siblings, and the family asset index. Logarithmically transformed LENA results (AWC, CTC, and CVC) are used in our regression models.

As our samples were randomly selected within counties and villages, according to previous studies (Gulliford, Ukoumunne and Chinn 1999; Agarwal, Awasthi and Walter 2005), we calculate the intraclass correlation coefficients (ICC) representing the proportion of the true total variation in the outcomes at county level or village level. The ICC representing the proportion of the true total variation in the AWC, CTC, and CVC at county level are respectively 0.0545, 0.0903, and 0.1514. The ICC representing the proportion of the true total variation in the AWC, CTC, and CVC at village level are respectively 0.3085, 0.3020, and 0.3802. Thus, the county fixed effects are used to control for the unobserved heterogeneity at the county level, and the standard errors are adjusted to account for clustering at the village level to improve statistical efficiency of the data used in this study when we conduct the regressions.

## Results

The descriptive statistics for the sample are shown in Table 1. The average age of the children in the sample was 24.5 months (SD = 2.07). Just over half (53%) of the sample was male. In terms of family characteristics, mothers were the primary caregivers in 74% of households, with the paternal grandmother being the primary caregiver in nearly all of the remaining households (data not shown). A little over half of mothers and fathers (66% and 53%, respectively) had completed middle school or above. Each household contained an average of two adults, and 26% of families (10/38) had multiple children. Finally, 63% of the fathers had lived at home for the majority of the past year.

Table 2 describes the LENA outcomes and CDI score. The average AWC for the sample was 13,428 (SD = 6,058), the average CTC was 559 (SD = 267), the average CVC was 2,140 (SD = 737), and the average CDI score was 45 (SD = 25). When we group the sample into the upper and lower 50% of each count, we find additional variation. The upper 50% of AWC had an average of 17,847 (SD = 5,436) while the lower 50% was almost half that at 9,010 (SD = 2,160). The average count of the upper 50% of CTC was 763 (SD = 209) and the lower 50% was less than half that at 354 (SD = 120). Following this same trend, the upper 50% of CVC had an average count of 2,755 (SD = 418) while the lower 50% had an average count of 1,526 (380). The CDI groupings also continued this trend, as the upper 50% had an average score of 65 (SD = 20) while the lower 50% had a score of 25 (SD = 9).

**Table 2:** Language Environment Analysis (LENA) outcome differences between urban and rural households

Groups		Rural northwestern China sample (N = 38)		Urban Shanghai sample (N = 22)	
		Obs	Mean (SD)	Obs	Mean (SD)
		(1)	(2)	(3)	(4)
(1)	Full sample AWC	38	13,428	22	21,098
			(6,058)		(7,693)
(2)	Upper 50% AWC	19	17,847	11	27,035
			(5,436)		(6,007)
(3)	Lower 50% AWC	19	9,010	11	15,160
			(2,160)		(3,261)
(4)	Full sample CTC	38	559	22	751
			(267)		(287)
(5)	Upper 50% CTC	19	763	11	986
			(209)		(203)
(6)	Lower 50% CTC	19	354	11	515
			(120)		(103)
(7)	Full sample CVC	38	2,140	-	-
			(737)		-
(8)	Upper 50% CVC	19	2,755	-	-
			(418)		-
(9)	Lower 50% CVC	19	1,526	-	-
			(380)		-
					-
(10)	Full sample CDI	38	45	-	-
			(25)		-
(11)	Upper 50% CDI	19	65	-	-
			(20)		-
(12)	Lower 50% CDI	19	25	-	-
			(9)		-

Source: Column 1 (“Rural northwestern China sample”) is from the authors’ survey. Column 2 (“Urban Shanghai sample”) is from Zhang et al. 2015.

Note: This table utilized data from urban Shanghai (Zhang et al., 2015). According to Zhang et al. (2015), volunteer parents of 22 children aged 3 to 23 months were recruited for their Shanghai sample. To recruit the participants, the Shanghai study team used flyers, emails, and word of mouth. A total of 22 participants were selected to record language development based on age group balance. Families provided daylong, in-home audio recordings using LENA. Three LENA recordings were collected over a 2-week period for each family to provide a stable estimate of the home language environment. Audio samples for the validation analyses were randomly drawn from one recording per family. The standard recording period was 16 h but given variability across families with respect to start and end times the Shanghai study restricted the potential sampling range to the 12 h between 9 am to 9 pm.

Table 2 also compares AWC and CTC between urban and rural households. For the AWC, the urban Shanghai sample (Zhang et al., 2015) had an AWC of 21,098, which was 7,670 greater than that of our rural sample (13,428), though the children in the Shanghai sample were distinctly younger than those in our sample [52]. Additionally, the gap in AWC between rural and urban families was largest in the below-median group, as rural families (9,010) lagged behind urban families (15,160) by 6,150 words. For the CTC, the CTC of the urban sample was 751, which was higher than that of our rural sample (with an average CTC of 559), though again the children in the Shanghai sample were distinctly younger than those in our sample. We similarly find the largest gap in conversational turns for families in the below-median group, with rural families (354) lagging behind urban families (515) by 161 turns. Finally, we were unable to compare CVC and CDI, as this data was unavailable in the urban dataset.

We next present multivariate simple linear correlations between child and family characteristics and our three indicators of the home language environment (Table 3). Surprisingly, these tests find no significant correlations between family characteristics and these three measures of the home language environment. While this is indeed a surprising result, the multivariate multiple linear regression analysis does find significant correlations.

**Table 3:** Multivariate simple linear regression analysis: Correlations between Language Environment Analysis (LENA) outcome measures (AWC, CTC and CVC) and family characteristics

VARIABLES		lnAWC	lnCTC	lnCVC
		(1)	(2)	(3)
(1)	Age of child (months)	-0.02	0.00	0.04
		(0.04)	(0.04)	(0.04)
(2)	Sex (1 = boy)	0.06	0.20	0.13
		(0.14)	(0.22)	(0.17)
(3)	Age of mother (years)	0.01	0.04	0.03
		(0.03)	(0.03)	(0.02)
(4)	Maternal education level (1 = completed middle school or above)	0.09	0.04	-0.12
		(0.26)	(0.27)	(0.14)
(5)	Mother is the primary caregiver (1 = yes)	-0.18	-0.08	0.02
		(0.13)	(0.22)	(0.12)
(6)	Number of adults in the household	0.13	0.09	0.00
		(0.05)	(0.10)	(0.10)
(7)	Whether there are siblings at home (1 = yes)	-0.25	-0.27	-0.12
		(0.21)	(0.36)	(0.15)
(8)	Paternal education level (1 = completed middle school or above)	0.29	0.17	-0.01
		(0.23)	(0.20)	(0.14)
(9)	Father lived at home during most of the last year (1 = yes)	-0.04	-0.02	-0.00
		(0.14)	(0.18)	(0.14)
(10)	Asset index (PCA score)	0.00	0.05	-0.02
		(0.06)	(0.08)	(0.04)
(11)	Observations	38	38	38

Source: Authors’ survey.

Standard errors in parentheses; standard errors were adjusted at the village cluster level. In our multivariate simple linear regressions, to control the family-wise error rate, we used the Bonferroni Correction to adjust the α value used to assess significance. The new α value is *p < 0.005

Table 4 contains the relationships between child and family characteristics and the home language environment, obtained by multivariate multiple linear regression. No significant associations between child/family characteristics and CTC/CVC were found in the multiple models, which is consistent with results from the simple regressions. Better educated fathers (p = 0.024) and more adults in the household (p = 0.026) were significantly associated with higher AWC after controlling for possible covariates.

**Table 4:** Multivariate multiple linear regression analysis: Correlations between Language Environment Analysis (LENA) outcome measures (AWC, CTC and CVC) and family characteristics

VARIABLES		lnAWC	lnCTC	lnCVC
		(1)	(2)	(3)
(1)	Age of child (months)	0.00	0.03	0.05
		(0.07)	(0.07)	(0.04)
(2)	Sex (1 = boy)	0.05	0.03	0.06
		(0.15)	(0.21)	(0.14)
(3)	Age of mother (years)	0.01	0.06	0.04
		(0.03)	(0.04)	(0.03)
(4)	Maternal education level (1 = completed middle school or above)	-0.18	0.00	-0.00
		(0.32)	(0.41)	(0.27)
(5)	Mother is the primary caregiver (1 = yes)	-0.34	-0.17	0.02
		(0.17)	(0.21)	(0.13)
(6)	Number of adults in the household	0.24*	0.13	0.02
		(0.10)	(0.13)	(0.10)
(7)	Whether there are siblings at home (1 = yes)	-0.18	-0.50	-0.35
		(0.33)	(0.51)	(0.25)
(8)	Paternal education level (1 = completed middle school or above)	0.43*	0.08	-0.05
		(0.17)	(0.17)	(0.22)
(9)	Father lived at home during most of the last year (1 = yes)	-0.22	-0.02	0.08
		(0.20)	(0.21)	(0.14)
(10)	Asset index (PCA score)	-0.03	0.10	0.02
		(0.10)	(0.15)	(0.07)
(11)	Observations	38	38	38
(12)	R-squared	0.39	0.33	0.35

Source: Authors’ survey.

Notes: In our multivariate multiple linear regressions, the county fixed effects are used to control for the unobserved heterogeneity at the county level.

Standard errors in parentheses; standard errors were adjusted at the village cluster level.

** p < 0.01, * p < 0.05

Figures 1 and 2, and 3 show the correlations between CDI score and AWC, CTC, and CVC, respectively. We find no significant correlation between CDI score and CVC, despite an upward trend. We find a positive relationship between CDI score and both AWC (P-value = 0.027) and CTC (P-value = 0.002). We do not find a significant correlation between CDI and CVC (P-value = 0.072), however.

## Discussion

In this paper we present the preliminary findings using the LENA system to measure the home language environment of young children from poor rural communities in China. We find an average AWC of 13,428 words per day among our sample of 20 to 27 months old children, however the variation in this count was quite large, with a standard deviation of 6,058. The CTC among our sample population was 559 per day, with a standard deviation of 267, which also indicated quite large variation. The average CVC among our sample population was 2,140 per day, with a relatively smaller but still large variation of 737 vocalizations. In comparison to other China samples, we find that the home language environment of our rural sample falls far behind that of urban households. Also, we find few correlations between sample characteristics and language environment measures, as only having a better educated father and having more adults in the household were significantly correlated with higher AWC, while the mother being the primary caregiver was significantly correlated with lower AWC. Though the literature on the link between the home language environment and cognitive development is mixed [7–9, 40, 72], we do find a positive correlation between the home language environment and children’s language abilities as measured by the CDI.

Contextualizing our findings by comparing the measured AWC and CTC of our rural sample to those of a sample of urban children living in Shanghai, we find that our rural sample falls far behind in both AWC and CTC [60]. This urban sample comes from a more developed setting, as evidenced by the fact that the average wage in Shanghai is twice that of Shaanxi [16]. Additionally, the education level of Shanghai residents is generally high; a large share of individuals with young children have tertiary levels of schooling [73]. It should be noted however, that we cannot directly compare the two samples, as the children in the Shanghai sample are distinctly younger than those of our sample, by about one year on average. Because the Shanghai sample is younger (and typically CTC grows over the first years of a child’s life [30]), if there are differences in adult words and conversational turns (and the Shanghai word counts and conversational turns are higher than the Shaanxi counts), these differences should be considered as lower bounds. In fact, even considering the measured gaps are lower bounds, the comparison between the two samples reveals drastic differences.

The urban Shanghai sample had an AWC that was 57.1% higher than our rural sample, amounting to a daily word gap of over 7,670 words. By age four, this could amount to a gross total gap of millions of words between urban and rural families. Due to the magnitude of this gap, we believe this is one of the primary differences between urban and rural home language environments in China. For the CTC, this difference is made especially clear when considering the natural changes in CTC as children age. In a healthy population, CTC increases by about 29.4 conversational turns per month between the ages of 13 and 27 months [30]. Using this figure to extrapolate the data from the Shanghai sample, we can estimate a 2-year-old age-adjusted CTC that is much higher among urban households (1,162) than it is for our own rural sample.

In comparing the home language environment between the rural and urban samples, we also find that the gap is largest for families in the bottom halves of the AWC and CTC distributions. For example, the gap in AWC between rural and urban families was largest in the below-median group: rural families lagged behind urban families by 68.3%. Even without the age-adjustment of the CTC for the Shanghai sample described above, we similarly find the largest gap in conversational turns for families in the below-median group, with rural families lagging behind urban families by around 45.5%. This suggests that while rural families across the spectrum are reporting poorer home language environments than are urban families, the gap with urban families widens even further among families who are already doing relatively poorly compared with rural families. Despite the age differences between rural and urban samples, our findings suggest that these drastic differences between urban and rural samples largely align with previous literature and present a serious problem, as has been noted in the discussion of the 30-million word gap by Golinkoff et al. (2019) [74].

When comparing our findings to those of other non-Western studies of the home language environment, we find that our measures of the home language environment are quite similar to those reported in the literature. In a study from South Korea [50] the measured AWC was 14,053 and CTC was 377. Given that these children were 10 months old on average, and that in a healthy population CTC increases by about 29.4 conversational turns per month between 13 and 27 months [30], we can extrapolate that the CTC of a 24-month old would be 788. We find that, while these scores are slightly ahead of those of our own sample, this gap is not meaningfully large. When we look at a study carried out in Senegal [51], whose measured CTC and CVC, after extrapolating hourly data into a 12-hour total, were 654 and 2,640, respectively, we similarly find that, while slightly higher than our own, the score differences between the sample from Senegal and our sample are not substantial. We believe that much of the variations found between non-Western samples are due to differences in beliefs and characteristic factors. For instance, in the Senegal study [51], cultural traditions and beliefs may discourage parents from verbally engaging with their young children and very low levels of education may hinder parent-child communication. In the study from South Korea [50], however, sample parents had very high levels of education and wealth compared to both our study and the that of Weber, Fernald, and Diop (2017) [51]. Despite these cultural variations, that our results are similar to that of other non-Western studies is seemingly surprising.

We find little evidence that the home language environment is worse in certain types of homes or with certain types of children. In this sense, our paper deviates from literature that shows that families with better educated mothers [24, 75] and families with daughters [24] typically provide more diverse home language environments to their children. Instead, our results are consistent with an interpretation that large variations exist in the rural home language environment, and that the language development of many young children is suffering because of this. Despite the fact that we do not find many significant correlations between child or family characteristics and the home language environment, the variations in the home language environment that we do find are troubling. Previous research has noted the importance of the home language environment for the development of children [7–9, 20], and thus large variation is a significant problem. In the context of rural China, these results may not be surprising. Studies that have looked at the knowledge base of rural Chinese parents have found a low understanding of the need for child stimulation at home [9]. In this way, China may be a victim of its own economic success. Only one generation ago, China had one of the highest global poverty rates, and a majority of its population were subsistence farmers [76]. In such conditions, keeping children safe and making them physically strong was a much higher priority than providing a cognitively stimulating or linguistically diverse environment. Evidence shows that even today, rural families still have limited access to reliable sources of information about the importance of providing children with stimulating home environments [77].

Overall, our findings from the non-Western cultural context of rural China are roughly consistent with findings from Western settings, however some disparities in the explanations for variation exist. There are large variations within the sample, which is fully consistent with the observations in Weisleder & Fernald (2013) [26]. Compared to results from a similar age group (18–24 months old) sample in the United States from Gilkerson et al. (2018) [35], our measured AWC (13,428) is actually 1,660 greater than their own (11,768). Our measured CTC (559) and CVC (2,140) were slightly higher and lower than the CTC and CVC (519 CTC and 2,152 CVC) of the 24 month old sample from Gilkerson & Richards (2008) 78 by 40 CTC and 12 CVC, respectively [78]. We find that the home language environment is directly and significantly correlated with child language outcomes. Within our sample, we are unable to identify either child- or family-level factors that correlate with the diversity of the home language environment, a finding that suggests a consistent pattern of behavior among rural families of all types. This deviates from findings from Western settings, which tend to find that wealthier and better educated families provide their children with more diverse home language environments [24, 41, 75].

We acknowledge several limitations of this study. First, the sample size is relatively small, which we cannot rule out as being a factor behind not finding statistically significant correlations between our LENA outcomes and child or family characteristics. More research of the home language environment using a larger sample size that includes different subpopulations (including rural and urban) is needed to better identify whether specific types of families or children are more at risk. Second, our findings are quite different from findings from non-Western cultural settings, and we did not engage in systematic study of specific cultural factors that may be shaping behaviors. Third, despite its widespread use in assessing the language environments of children, the LENA measurements we use in this study (AWC, CTC and CVC) are not entirely accurate, especially CTC [40, 72]. Further research should be conducted on the variation between non-Western home language environments, and the reasons behind this variation. More research is also required to better understand the reasons—cultural or otherwise—behind the low levels of language engagement that we observe among the rural Chinese population. The exploration of interventions involving parental coaching may also be valuable, as they have been shown to increase CTC and infant language development [42].

## Electronic supplementary material

Below is the link to the electronic supplementary material.

Supplementary Material 1

## Data Availability

The data that support the findings of this study are available from the corresponding author upon reasonable request. The data are not publicly available due to privacy or ethical restrictions.

## Footnotes

1 In rural China, where there are essentially no childcare services (Qiao et al., 2015), a “normal day” typically consists of the mother or grandmother of the household rising around 6:00 am to perform house chores such as cleaning and cooking breakfast. Afterwards, the primary caregiver takes care of the children at home or at a neighbor’s house until lunch. Then, while the caregiver prepares dinner, a child might take a nap in the afternoon or play by themselves or with other children. After dinner, the caregiver will prepare the child for bed by telling them stories or watching television together. [Qiao, F., Rozelle, S., Zhang, L., Yao, Y., & Zhang, J. (2015). Impact of Childcare and Eldercare on Off-farm Activities in Rural China. China & World Economy, 23(2), 100–120. https://doi.org/10.1111/cwe.12109]
