# Supplementary material for: The home language environment in rural China: variations across family characteristics
Source: BMC Public Health. 2023 Feb 16;23:354. doi: 10.1186/s12889-023-15245-2 (PMC9936727; doi:10.1186/s12889-023-15245-2)
Supplement: Supplementary file 1 — Supplementary Material 1 [file 12889_2023_15245_MOESM1_ESM.docx]

| **Appendix Table 1. Mean differences and correlations between human rater counts and Language Environment Analysis (LENA) estimates** | | | | | | | | | |
| --- | --- | --- | --- | --- | --- | --- | --- | --- | --- |
| Rater measure | LENA measure | N | Rater  M (SD) | LENA  M (SD) | Difference*  M (SD) | p | r | p (r) | R^2^ |
| Chinese characters | AWC | 13 | 594 (217) | 398 (171) | 196 (155) | **<.001** | .56 | **.01** | .50 |
| SDM words | AWC | 13 | 400 (156) | 398 (171) | 3 (95) | .92 | .92 | **<.001** | .70 |
| Conversation turns | CTC | 13 | 31 (13) | 27 (8) | 5 (8) | .06 | .50 | **<.001** | .69 |
| Child vocalizations | CVC | 13 | 97 (48) | 92 (25) | 5 (36) | .65 | .36 | **.01** | .47 |
| Note. SDM = Shaanxi dialect and Mandarin; AWC = adult word count; CTC = conversational turn count; CVC= child vocalizations count; M = mean; SD = standard deviation. Bold values indicate p values greater than alpha = .05.  *Difference values reflect rater-based counts minus LENA-based counts. | | | | | | | | | |

**Appendix Figure 1. Kernel density distribution of Adult Word Count**

**
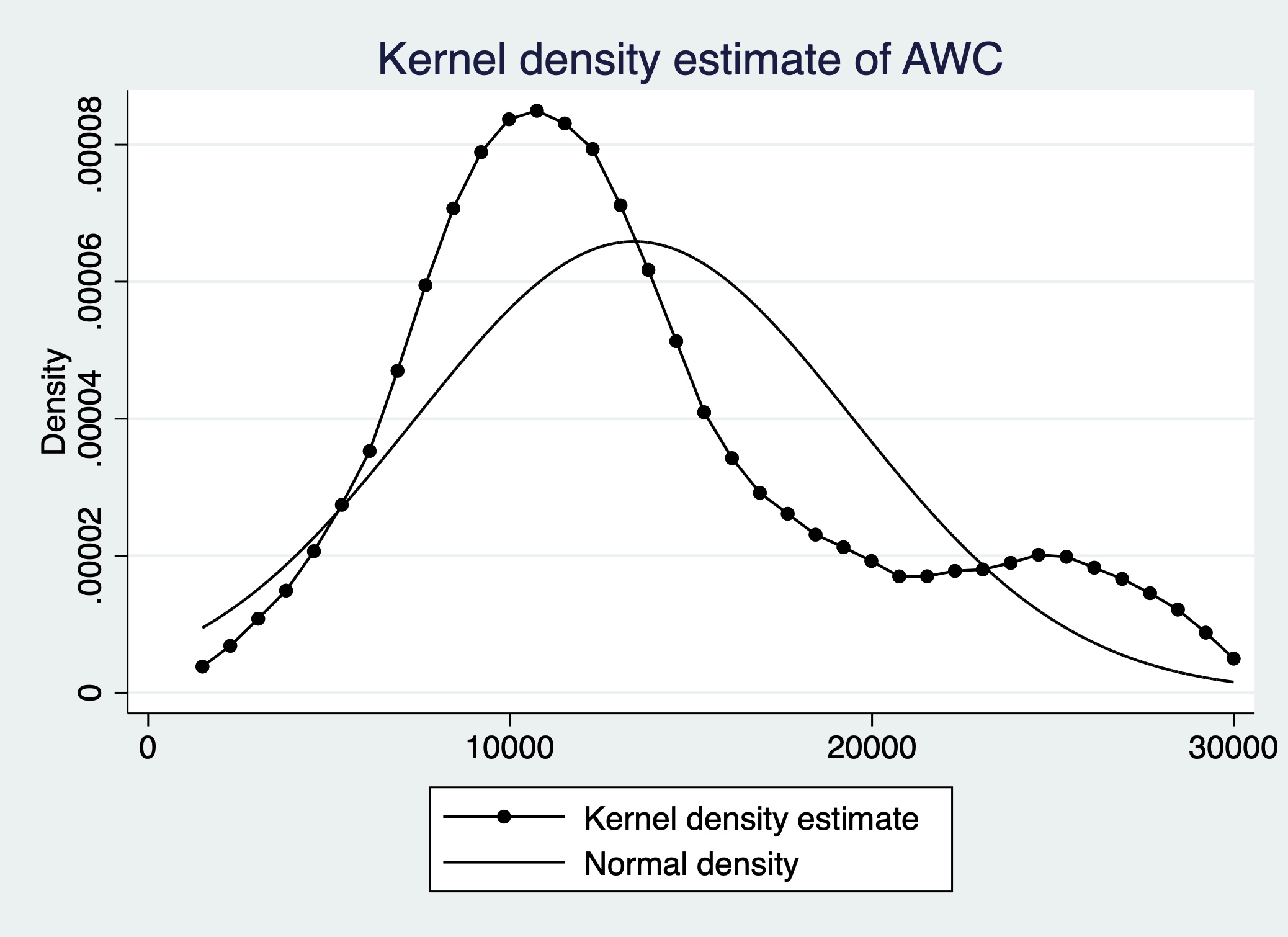
**

**Appendix Figure 2. Kernel density distribution of Conversational Turns Count**

**
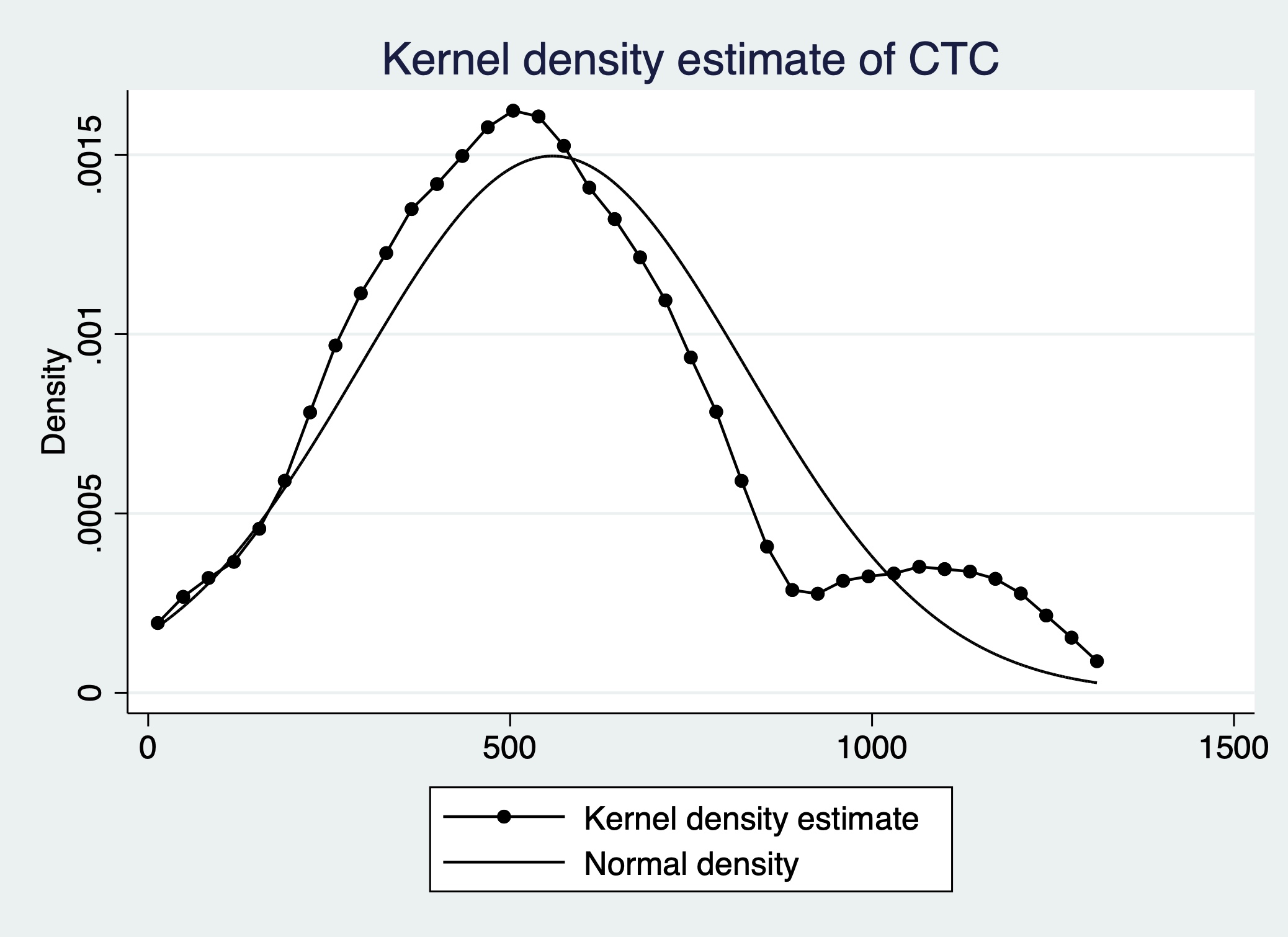
**

**Appendix Figure 3. Kernel density distribution of Child Vocalization Count**

**
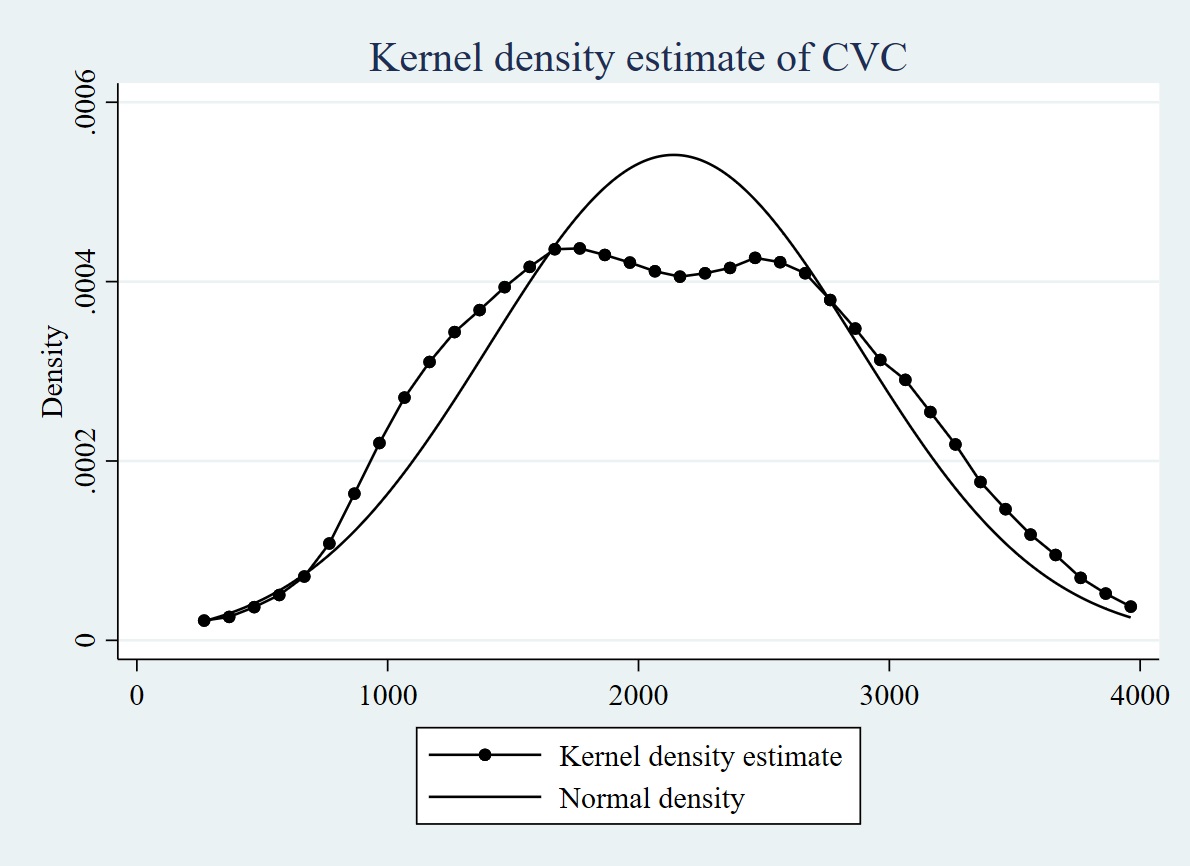
**

**Appendix Protocol:**

**Guidelines for Validating LENA Adult Words, Conversational Turns, and Child Vocalizations in the Chinese Mandarin Shaanxi dialect based on Gilkerson et al. (2015) and Gilkerson & Richards (2020)**

**Step 1: Recording file selection**

First, we use Stata 16.1 (StataCorp, College Station, Texas, USA) to randomly choose 13 households from 38 households (1/3). Recordings of 13 households should reflect environmental conditions typical of the research focus and should not contain long periods when the child is not wearing the recorder. In addition, recordings of 13 households should be at least one daylong recording (10 hours minimum). If families have provided multiple recordings, it is best to eliminate the first day from the validation set, as it may be less representative of families’ naturalistic behaviors.

**Step 2: Audio segment selection**

Next, select a minimum of three 5-minute audio segments per file, representing periods of high, medium, and low interaction. More specifically, determine the total amount of Conversational Turns (CT) for each file, and then rank each 5-minute segment relative to the overall CT count. The purpose of focusing on high-activity turns regions is to ensure that there are enough turns in the coded segments to evaluate turn-taking. Then randomly choose:

a. One 5-minute segment from the top 10% of turns counts, excluding the highest;

b. One 5-minute segment from the middle 20% of turns counts;

c. One 5-minute segment from the bottom 10% of turns counts, excluding all zero counts.

Before coding segments, listen to each 5-minute segment to evaluate its content and context. Segments should be excluded if they represent relatively low likelihood (unrepresentative) events that are unusually noisy (e.g., a birthday party or sporting event), or activities in which turns cannot be confidently assessed (e.g., caregiver is on the phone, making it unclear whether vocal alternations represent turns from the point of view of a child). Do not exclude segments if they reflect higher likelihood events more representative of activities expected to occur relatively often throughout the day. The purpose of excluding samples at this stage is to avoid introducing outlier data that could unduly influence results in a small sample.

**Step 3: Counting adult words, conversational turns, and child vocalizations**

For the Adult Words, the rater annotates each segment identified as containing clear adult speech with two count values: the number of discrete Chinese characters and the number of Romanized pinyin words by which adult speech in the segment could be represented (see http://www. pinyin.info). Chinese characters are not letters but rather are a form of pictogram. In most cases, each character is equivalent to one spoken syllable, and spoken Chinese Shaanxi dialect and Mandarin words typically comprise between one and four syllables. Following standard word-formation rules in grouping characters to derive word boundaries, the rater counts full Adult Words (see http://www.pinyin.info/readings/zyg/rules.html). For example, 差不多cha bu duo has a character count of three but connotes a single word (*almost*), as does 为什么wei shen me (*why*). Grouping Chinese characters into words requires some degree of flexibility, so the rater generally reports the minimum number of words necessary to convey the meaning. Counting rules for different types of speech are as follows:

**Clear speech**: Words should be counted if the listener can clearly determine the word, regardless of how far away the listener might believe the child is from the speaker, how loud the speaker is, or whether the speech was directed to the child.

**Unclear speech:** If the listener hears an adult sentence or phrase in which some of the words are inaudible, count ONE word for each inaudible part regardless of its length, even if the listener guessed there was more than one word in the string. For example, if the listener hears: "ni xian zai qu xxx! Ni yi jing xxx, wo rang ni qu shui jiao!" the listener would count a total of 15 words. The assumption here is that since we do not know exactly how many words are in the inaudible segment (but we know there is at least one word based on the context), we give the speaker credit for one word.

**"Fillers"**: Vocal pauses in speech (e.g., er, um) are not counted as separate words.

**Interjections, exclamations:** If an adult makes a communicative “sound” that may not necessarily be called a word in the traditional sense but has meaning, listeners should count it as a word. For example, if the adult looks at a mess the child made and says "ya," count this as one word. Or if the parent says "eiyou" in surprise count each as one word.

**Vocal play:** If the parent makes a sound or long utterance that can be considered vocal play but has no discernible words, listeners should count the utterance as one word. For example, if the child says "Ba ba ba ba" and the adult repeats "Ba ba ba ba," count that string as one word.

**Singing and whispering:** Listeners should count any words the adult sings or whispers-in general, count any word that reasonably can be discerned.

**Overlapping speech**: If the listener hears words spoken at the same time as overlapping noise or other human speakers, the words should not be counted. The assumption here is that the sound signal the child is experiencing is distorted-since competing signals add to the parsing burden, we cannot be confident that the child is absorbing the linguistic information. Alternately, these words can be tallied separately.

**TV/Electronic Sounds:** Adult Words coming from a speaker of any sort (e.g., TV, radio, iPad) should NOT be included in the Adult Word Count.

In terms of Conversational Turns, CTs include only back-and-forth interactions between the child wearing the recorder and an adult in his/her environment. The child does not need to be speaking words to engage in interaction—if he/she makes a sound in response to a parent (or vice versa), this is a turn. Goos, growls, raspberries, vowels, quasi-vowels, nasals, etc. are all considered infant communicative sounds and should be included in turns. LENA does not include fixed signals (e.g., cries, screams, whimpers, laughs), which are considered instinctive reactions to the environment, or vegetative sounds (e.g., burps, breaths, coughs, etc.), so ignore these sounds when determining whether a turn has occurred.

**The 5 Seconds Rule:** If the child responds to an adult or an adult responds to the child within 5 seconds of the end of the utterance, one turn is counted. In addition, if an adult speaks, the child responds, and then the adult responds back (i.e., adult-child-adult), count only one turn. Each speaker segment can be included in only one turn -there is no double-dipping in LENA count. If the child responds to an adult after 5 seconds have passed, this should not be counted as a turn. Same applies if the adult responds to the child after 5 seconds have passed.

Adult-child-adult=1 turn

Child-adult-child=1 turn

Adult-child-adult-child=2 turns

**Interruptions**: If the parent is in the middle of a sentence and the child interrupts, count one turn (essentially, this is a response within zero seconds). Same applies if the adult interrupts the child.

**Whining:** If a child seems to be communicating words during a whine and the adult responds, then count it as a turn. Otherwise, whines without words or clear intentional communication should not be included in turn counts.

Regarding Child Vocalization (CV), CV estimates the number of any speech-like babbling or vocalizations within a child utterance cluster. Fixed signals (e.g., cries, screams) and vegetative noises (e.g., burping) were not count as vocalizations. For instance, if the child said "ba" or "bababababa" this is counted as one vocalization, whereas if the child said "bababa # baba," this is counted as two vocalizations. During the single-word period and the two-to-three-word combinations, a word is counted as one vocalization. Thus, if the child says "bababa# papa# mama", this is counted as three vocalizations.

References:

1. Gilkerson, J., Zhang, Y., Xu, D., Richards, J. A., Xu, X., Jiang, F., ... & Topping, K.2015. Evaluating language environment analysis system performance for Chinese: A pilot study in Shanghai. Journal of Speech, Language, and Hearing Research, 58(2), 445-452.
2. Gilkerson, J., & Richards, J. A. 2020. A Guide to Understanding the Design and Purpose of the LENA® System. (LENA Technical Report# 12).
